# Integration of Multiple Signaling Regulates through Apoptosis the Differential Osteogenic Potential of Neural Crest-Derived and Mesoderm-Derived Osteoblasts

**DOI:** 10.1371/journal.pone.0058610

**Published:** 2013-03-25

**Authors:** Shuli Li, Nathaniel P. Meyer, Natalina Quarto, Michael T. Longaker

**Affiliations:** 1 Hagey Laboratory for Pediatric Regenerative Medicine, Department of Surgery, Stanford University, School of Medicine, Stanford, California, United States of America; 2 Dipartimento di Scienze Biomediche Avanzate, Universita’ degli Studi di Napoli Federico II, Napoli, Italy; Ohio State University, United States of America

## Abstract

Neural crest-derived (FOb) and mesoderm-derived (POb) calvarial osteoblasts are characterized by distinct differences in their osteogenic potential. We have previously demonstrated that enhanced activation of endogenous FGF and Wnt signaling confers greater osteogenic potential to FOb. Apoptosis, a key player in bone formation, is the main focus of this study. In the current work, we have investigated the apoptotic activity of FOb and POb cells during differentiation. We found that lower apoptosis, as measured by caspase-3 activity is a major feature of neural crest-derived osteoblast which also have higher osteogenic capacity. Further investigation indicated TGF-β signaling as main positive regulator of apoptosis in these two populations of calvarial osteoblasts, while BMP and canonical Wnt signaling negatively regulate the process. By either inducing or inhibiting these signaling pathways we could modulate apoptotic events and improve the osteogenic potential of POb. Taken together, our findings demonstrate that integration of multiple signaling pathways contribute to imparting greater osteogenic potential to FOb by decreasing apoptosis.

## Introduction

Differences in the embryonic origin of mammalian bones composing the cranial vault have been established [1]. While the frontal bones of the calvarium are neural crest in origin, the parietal bones arise from paraxial mesoderm [1]. Our initial work unveiled that the different embryonic origin of calvarial bones influences their osteogenic potential and tissue repair both *in vitro* and *in vivo* cells [2], [3], [4], [5]. Further studies have ensued to elucidate the molecular biology responsible for orchestrating the process of controlled osteogenesis in these tissues. A multitude of signaling pathways have been assigned to play roles in the regulation of frontal and parietal bone osteogenic potential. For instance, we have demonstrated that the neural crest derived-frontal osteoblasts (FOb) are endowed with enhanced activation of endogenous canonical Wnt signaling compared to the paraxial mesoderm-derived osteoblasts (POb) and that this signaling contributes to the greater osteogenic potential of frontal bone cells [2]. Additionally studies also revealed that FOb, as well frontal bone tissues feature higher level of activation of all three FGF signaling pathways, Erk, Akt and PKC, compared to parietal bone and that express significantly more of FGFs pro-osteogenic ligands *fgf-2,* -*9* and -*18* and *fgf Receptor* -*1* -*2* and -*3*. [3], [4], [5]. Moreover, evidence obtained from both *in vitro* and *in vivo* studies indicated that this differential signaling plays a critical role in conferring greater osteogenic potential and tissue regeneration to frontal bones [3], [4], [5]. For example, calvarial defects in *fgf-9* and *fgf-18* haploinsufficient mice showed impaired calvarial healing in frontal bones to a level similar to that observed in parietal bone of CD1/wild type mice skull [5]. Certainly, delineating regional differences in osteogenic potential based on embryonic origin and determining the role of differential signaling in imparting superior osteogenic potential and bone repair is an appealing challenge.

Apoptosis, the process of programmed cell death, has been widely implicated to play roles in skeletal development, bone remodeling, turnover and regeneration [6], [7], [8]. For example, an *in vitro* study demonstrated that a higher differentiation status and bone-forming ability of osteoblasts is associated with low apoptosis [9]. Furthermore, excessive apoptotic activity was found to delay osteogenesis during development of mouse calvarial bone and sutures [7], [10].

Searching for additional molecular mechanisms implicated in the higher osteogenic capacity of frontal bone, the potential role for apoptosis stimulated our interest. In the current study we present evidence that in comparison to FOb, POb have higher apoptotic activity which negatively impacts their osteogenic potential. Moreover, our results indicate that the elevated apoptosis observed in POb is under orchestrated control of integrated signaling pathways displaying different levels of activation. Among them, TGF-β, BMP and canonical Wnt play important roles. Thus, the differential osteogenic potential and tissue regeneration establish neural crest-derived frontal bone and the mesenchymal-derived parietal bone as an excellent model for investigating specific signaling pathways and their connection with apoptosis and osteogenesis.

## Results

### Apoptotic Activity Inversely Correlates with the Osteogenic Potential of FOb and POb

**Figure 1:**
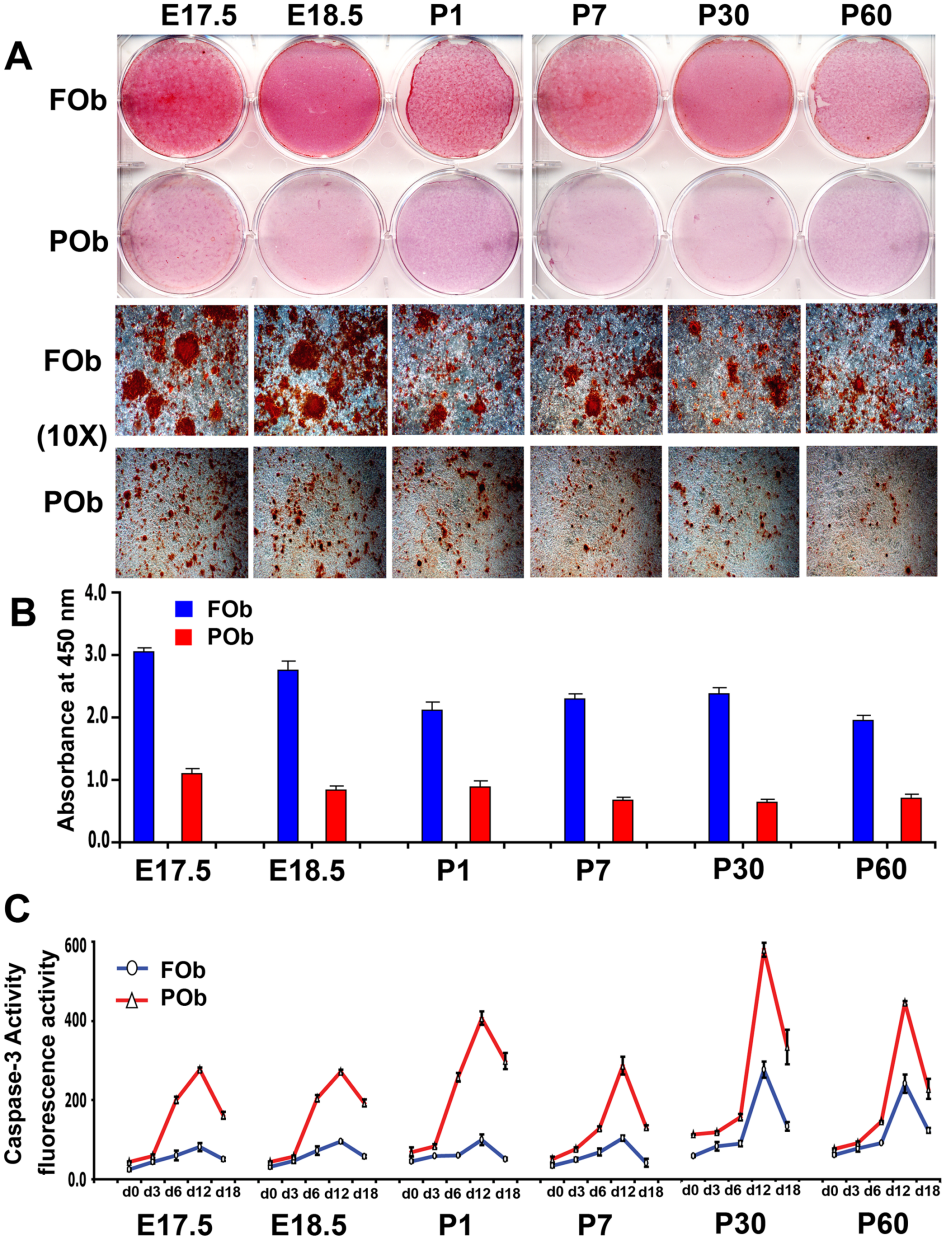
Apoptotic profile of FOb and POb cells undergoing to osteogenic differentiation. (A) Alizarin red staining of FOb and POb cells at differentiation day 21 shows striking differences between FOb and POb, with FOb cells having a more robust mineralization and larger bone nodules as seen in the lower panel (Magnification 10X). (B) Quantification of Alizarin red staining. (C) Time course of Caspase-3 activity performed during osteogenic differentiation of FOb and POb cells harvested from different mouse stages reveals significant higher apoptotic activity in POb cells with a peak at d12 of differentiation. Abbreviations: (E), embryonic; (P), postnatal.

The different osteogenic potential of FOb and POb can be identified in mice as early as embryonic stage 17.5, and persists throughout life-span as previously demonstrated [2], [3] and shown herein (**Figure 1A and B** **)**.

As first, to unveil potential differences in apoptotic activity between the two types of osteoblasts, we investigated apoptosis during the timing of their osteogenic differentiation. Cell lysates were collected at different time points and analyzed for caspase-3 activity, a key apoptotic protease. This assay detected significantly higher caspase-3 activity in POb than FOb at all time points analyzed, starting from day 0 prior differentiation (**Figure** **1C**). The apoptotic activity increased during differentiation reaching a peak by day 12, followed by a sharp decrease at day 18. This profile was consistent among all different mice stages. Apoptotic activity was also observed in FOb but the degree was significantly lower, however the trend was similar. Moreover, FOb and POb derived from late stages such as postnatal day 30 (P30) and day 60 (P60), showed overall higher apoptotic activity than earlier stages (E 17.5, E18.5 P1 and P7). The latter observation would suggest that age can influence apoptosis.

To gain direct evidence on the role of apoptosis in determining differences in the osteogenic potential between FOb and POb we performed an osteogenic assay in presence of the inhibitor of caspase-3 Ac-DEVD-CHO. Treatment with 0.05 µM of Ac-DEVD-CHO inhibited dramatically the caspase-3 activity while increasing osteogenic differentiation in POb to level similar to that of FOb (**Figure S1**). All together, the above results revealed the occurrence of higher apoptotic activity in POb compared to FOb, which inversely correlated with the osteogenic potential of FOb and POb, therefore, linking inhibition of apoptosis with increased osteogenic capacity of POb. These initial observations suggested that indeed, apoptosis might be one of the underlying mechanism(s) governing the different osteogenic potential between FOb and POb. Noteworthy, *in situ* apoptosis by TUNEL assay performed on coronal sections derived from frontal and parietal bone revealed a similar apoptotic pattern *in vivo* as well (**Figure S2**).

### Relationship between Endogenous Activation of TGF-β, BMP Signaling and Apoptosis in FOb and POb

Having unveiled significant differences in apoptotic activity between FOb and POb, next we sought to investigate the potential underlying signaling pathways.

**Figure 2:**
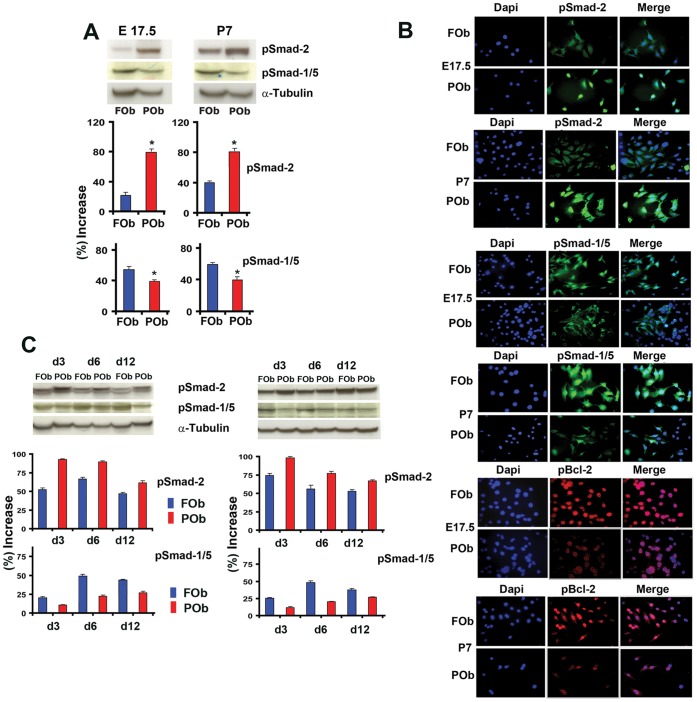
Differential activation of endogenous TGF-β and BMP signaling between FOB and POb cells. (A) Immunoblotting analysis using specific anti-phosphoSmad-2 and phoshoSmad-1/5 antibodies shows a more intense phosphorylation of Smad-2 in POb than FOb cells. In contrast, analysis with anti-phoshoSmad-1/5 antibody reveals a stronger staining in FOb. To assess for the total amount of endogenous Smad-2 and Smad-1/5 and to control for equal loading and transfer of the samples membranes were reprobed with anti-Smad-2, Smad-1/5 and anti-α-Tubulin antibodies. Histogram below represents quantification of phosphorylated Smad-2 and Smad-1/5 proteins obtained by Image J program. The relative intensity of each band was normalized to their respective α-Tubulin loading controls. (B) immunofluorescent staining using anti-phosphoSmads antibodies as above confirms the results obtained by immunoblotting analysis. Immunofluorescent staining using anti-phosphoBcl-2 antibody detects higher levels of the anti-apoptotic protein Bcl-2 in FOb compared to POb. Dapi nuclear counterstaining. (C) Immunoblotting analysis performed as above (A) showing that the differential activation of the two signaling pathways observed between FOb and POb cells is maintained throughout their osteogenic differentiation.

Among the several signaling pathways, TGF-β and BMP pathways have been reported to regulate apoptosis in addition to osteogenesis [11], [12], [13], [14]. Therefore, we investigated the status of endogenous activation of these two signaling pathways by analyzing the extent of phosphorylation of Smad-2 and Smad-1/5, the main down-stream signaling molecules of active TGF-β and BMP signaling pathways, respectively. To investigate the endogenous phosphorylated Smad-2 and Smad-1/5 proteins, we performed immunoblotting analysis using specific antibody for pSmad 2 and pSmad 1/5, focusing on E17.5 and P7 FOb and POb cells. As shown in **Figure 2A**, there was a consistent differential activation of both TGF-β and BMP pathways between FOb and POb either at embryonic or postnatal stages, as indicated by phosphoSmad-2 and phosphoSmad-1/5 proteins. Specifically, there was higher activation of TGF-β signaling in POb than FOb, and viceversa, BMP signaling was more activated in FOb than POB. These data were corroborated by indirect immunofluorescent analysis performed on FOb and POb cells using the same antibodies as above, which detected a more intense nuclear staining for phosphoSmad-2 in POb and for phosphoSmad-1/5 in FOb, respectively (**Figure 2B** **).** Interestingly, immunofluorescent analysis of the anti-apoptotic molecule Bcl-2, showed a pattern inversely correlating with that of phosphoSmad-2, while directly correlated with phosphoSmad-1/5 staining (**Figure 2B**). We observed intense nuclear staining of phosphorylated Bcl-2 in FOb which were strongly positive for nuclear phosphoSmad-1/5 staining and less for phosphoSmad-2. In contrast, in POb the Bcl-2 fluorescent staining was less intense and mainly cytoplasmic.

Thus, in FOb and POb there is a differential activation of TGF-β and BMP signaling, with more active TGF-β signaling in POb overlapping with less pBcl-2 expression, whereas FOb showed higher levels of active BMP signaling and pBcl-2 nuclear staining. These results suggest a relationship between the differential activation of TGF-β and BMP signaling pathways and apoptotic events. Moreover, profiling of phosphoSmad-2 and phosphoSmad-1/5 during osteogenic differentiation revealed that the differential activation of endogenous TGF-β and BMP signaling was sustained throughout differentiation (**Figure 2C**).

### Decreased Apoptosis in Differentiating POb Upon Inhibition of TGF-β Signaling

**Figure 3:**
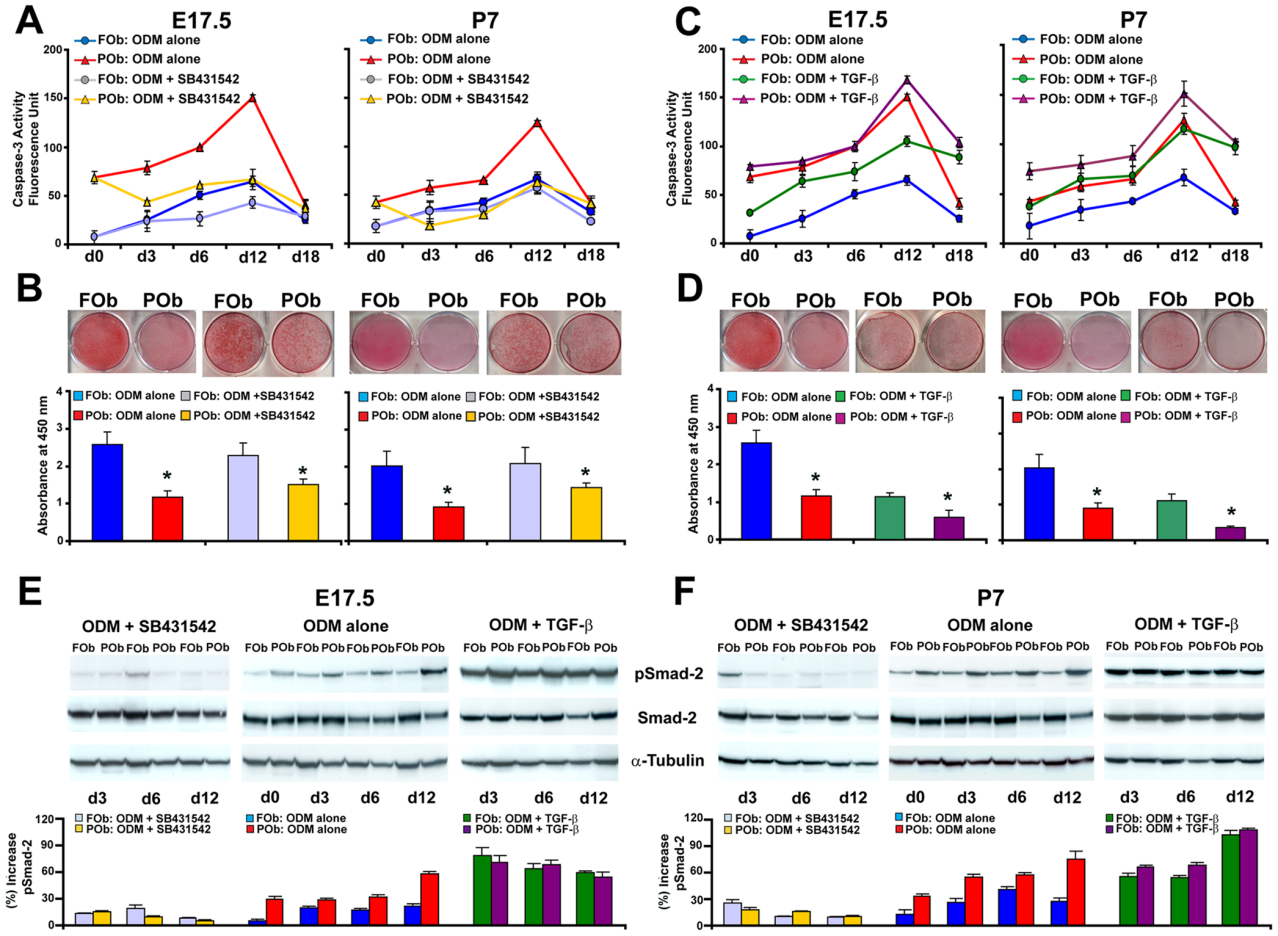
Inhibition of TGF-β signaling decreases the apoptotic activity of POb cells. (A) Time-course of Caspase-3 activity performed on FOb and POb cells undergoing osteogenic differentiation in presence of 10 µM SB431542, a specific inhibitor of TGF-β signaling reveals significant decrease of apoptotic activity in treated POb to a level similar to that of untreated FOb cells. A slight decrease of apoptosis is also observed in SB431542 treated FOb cells. (B) Alizarin red staining at day 21 showing that SB431542 treatment increases mineralization of extracellular matrix in POb. Alizarin red staining quantification (lower panel). (C) Time-course of Caspase-3 activity performed on FOb and POb cells undergoing osteogenic differentiation in presence of 10 ng/ml TGF-β1. This treatment increases significantly apoptosis in both FOb and POb cells. (D) Alizarin red staining showing that TGF-β1 treatment decreases mineralization of the extracellular matrix in both cells types. Alizarin red staining quantification (lower panel). (E) and (F) Immunoblotting analysis using specific anti-phosphoSmad-2 antibody to validate the effective inhibition and/or activation of TGF-β signaling upon specific treatments. To control for equal loading and transfer of the samples membranes were reprobed with anti-Smad-2, anti-α-Tubulin antibodies.

To explore in detail potential relationship between the apoptotic activity and differential activation of TGF-β signaling in FOb and POb we analyzed the effect that either inhibition or activation of TGF-β signaling could have on apoptosis in FOb and POb undergoing to osteogenic differentiation. To this aim, a differentiation assay was performed either in presence of exogenous recombinant TGF-β1 protein at a concentration of 10 ng/ml, or 10 µM of the specific inhibitor of TGF-β signaling SB 431542 [15]. Untreated cells were used as control. Upon treatment of FOb and POb with SB 431542, we observed a significant decrease of apoptosis in POb with a profile mirroring that of untreated FOb cells (**Figure 3A**), and increased mineralization of POb extracellular matrix (**Figure 3B**). SB 431542 treatment lowered apoptosis also in FOb (**Figure 3A**). Conversely, treatment with exogenous TGF-β protein increased apoptotic activity in both FOb and POb. The apoptotic level in treated FOb mirrored that of untreated POb cells (**Figure 3C**). TGF-β treatment inhibited mineralization of extracellular matrix as indicated by Alizarin red staining (**Figure 3D**). The proper activation and/or inhibition of TGF-β signaling in treated cells was assessed by immunoblotting analysis of phosphorylated Smad-2 (**Figures 3E and 3F**).

### Exogenous Activation of BMP Signaling Pathway Decreases Apoptosis in POb

**Figure 4:**
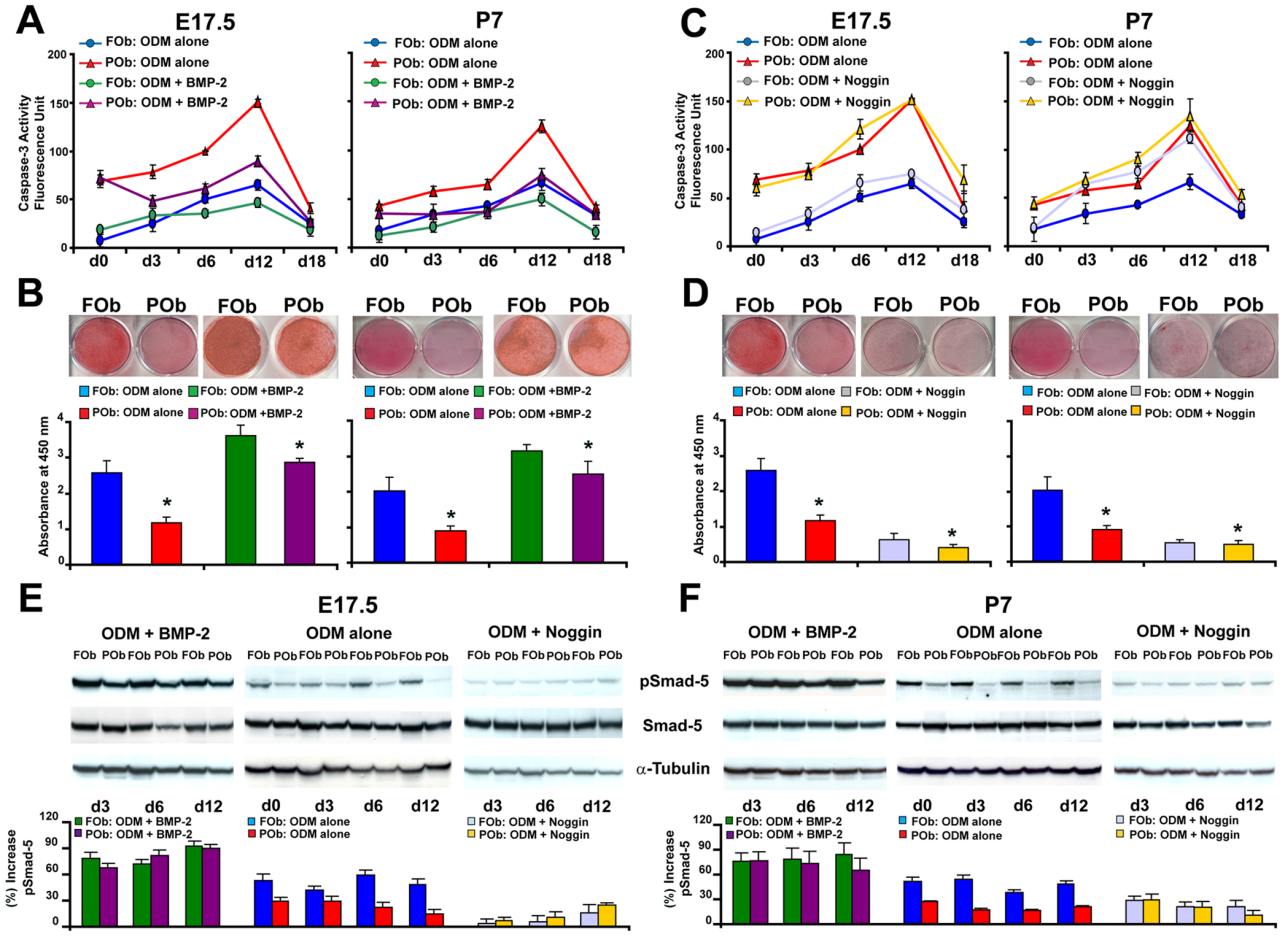
Exogenous activation of BMP signaling decreases apoptosis in POb cells. (A) Histogram illustrating a time-course of Caspase-3 activity performed on FOb and POb cells undergoing osteogenic differentiation with or without exogenous BMP-2 (200 ng/ml). BMP-2 treatment decreases apoptosis markedly in POb cells compared to untreated POb, a lower apoptotic activity is also observed in BMP-2 treated FOb as compared to untreated FOb cells. (B) Alizarin red staining and its quantification (lower panel) showing higher levels of mineralization in BMP-2 treated cells. (C) Profile of Caspase-3 activity in FOb and POb cells undergoing osteogenic differentiation with or without 200 ng/ml of noggin, inhibitor of BMP signaling, reveals that this treatment upregulated apoptosis with major effect on P7 osteoblasts (D) Noggin treatment inhibits dramatically mineralization of the extracellular matrix on both FOb and POb treated cells, as assessed by Alizarin red staining. The lower panel represents quantification of Alizarin red staining. (E) and (F) immunoblotting analysis with specific anti-phosphoSmad-1/5 antibody to validate the effective inhibition and/or activation of BMP signaling upon specific treatments. To control for equal loading and transfer of the samples membranes were reprobed with anti-Smad-1/5 and anti-α-Tubulin antibodies.

Next, we investigated whether BMP-2 signaling could play a role in apoptosis of FOb and POb undergoing differentiation. Using a similar experimental approach as above, the cells were treated either with exogenous BMP-2 protein or with noggin, an inhibitor of BMP signaling. The results obtained from this set of experiments were just the opposite of what previously obtained by treatment with either TGF-β1 or SB 431542. As shown in **Figure 4A**, addition of BMP-2 at concentration of 200 ng/ml significantly decreased apoptosis in POb undergoing to differentiation therefore, promoting a more robust osteogenic differentiation than untreated POb, as indicated by Alizarin red staining and its quantification (**Figure 4B**). In contrast, when cells were treated with 200 ng/ml of noggin, we observed increased apoptosis in both FOb and POb with a major effect on the P7 osteoblast population (**Figure 4C**). The increase in apoptosis was paralleled by a dramatic decrease in mineralization of extracellular matrix as shown by Alizarin red staining (**Figure 4D**). Immunoblotting analysis for phosphoSmad-1/5 confirmed the effective activation and/or inhibition of BMP signaling in treated FOb and POb (**Figures 4E and 4F**).

### Enhanced Endogenous Activation of Wnt Signaling Protects FOb from Apoptosis

**Figure 5:**
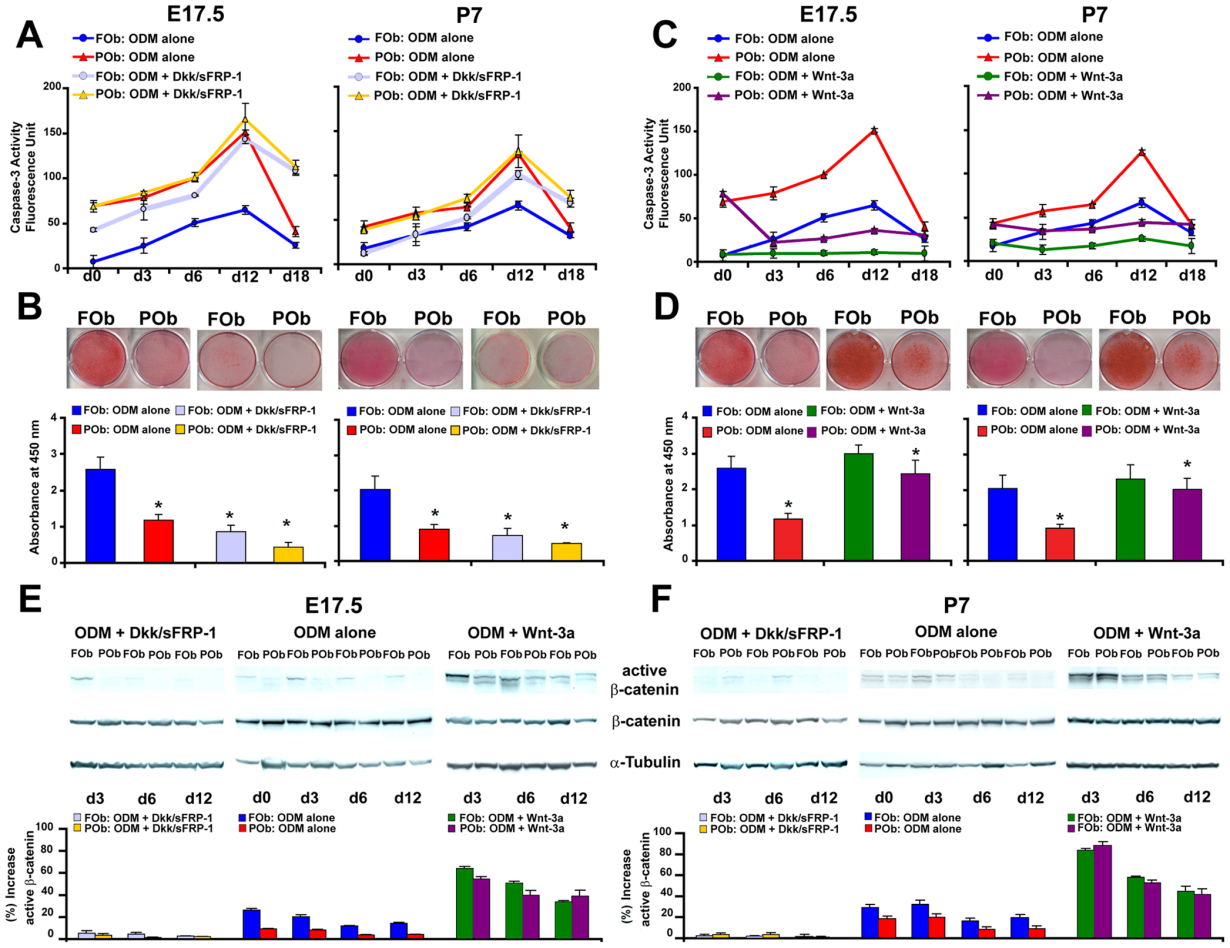
Enhanced activation of endogenous Wnt signaling in FOb and exogenous activation of Wnt signaling in POb cells protects from apoptosis. (A) Time-course Caspase-3 activity performed on FOb and POb cells undergoing osteogenic differentiation with or without Dkk and sFRP (150 ng/ml of each), as illustrated by histogram this treatment upregulates the apoptotic activity in treated FOb cells (B) The same treatment reduces dramatically mineralization of the extracellular matrix in both cell types. (C) Contrary, treatment with Wnt3a (50 ng/ml) robustly decreases apoptosis in POb cells compared to untreated cells. Wnt3a has a similar effect also on FOb cells. (D) Mineralization of the extracellular matrix detected by Alizarin red staining reveals a significant increase of osteogenesis in treated POb cells. (E) and (F) Immunodetection of active/unphosphorylated β-catenin showing the effective inhibition and/or activation of Wnt signaling upon specific treatments.

Our previous study indicated that FOb, as well frontal bone tissue, have enhanced activation of endogenous canonical Wnt signaling [2]. Because it has been suggested that Wnt signaling plays a role in apoptosis [16], [17], we investigated whether the enhanced activation of Wnt signaling in FOb is anti-apoptotic. To verify our hypothesis, we treated FOb and POb with two inhibitors of Wnt signaling Dkk1 and sFRP-1. Caspase-3 activity measured at different time points of osteogenic differentiation indicated that inhibition of Wnt signaling dramatically increased apoptosis in FOb compared to untreated FOb, (**Figure 5A**). Interestingly, inhibition of Wnt signaling in POb did not affect the apoptotic activity. This might be due to the poor activation of endogenous canonical Wnt signaling [2], and therefore, lack of target for the Dkk1 and sFRP-1 inhibitors. This hypothesis is supported by the observation that exogenous Wnt3a protein strongly decreased apoptosis in POb, while activating β-catenin (**Figure 5C**). The response of POb to Wnt3a stimulation was significantly higher than FOb (**Figure 5C**), as consequence of minimal endogenous active canonical Wnt signaling in POb. Conversely, treatment with inhibitors had a dramatic effect on FOb due to elevated level of endogenous active canonical Wnt signaling [2]. The elevated apoptotic activity observed in FOb treated with inhibitors was associated with a severe inhibition of mineralization (**Figure 5B**). Inhibition of osteogenesis was also observed in treated POb. Conversely, a complementary experiment using Wnt3 protein (50 ng/ml) demonstrated that activation of Wnt signaling inhibited apoptosis in cells undergoing to differentiation (**Figure** **5C**). Wnt3a treatment also induced a more robust osteogenic differentiation in POb cells (**Figure 5D**). Finally, the proper activation and/or inhibition of canonical Wnt signaling in treated cells was assessed by immunoblotting analysis of unphosphorylated/active β-catenin (**Figures 5E and 5F**).

## Discussion

This study highlights new features delineating further differences between neural crest-derived and mesoderm-derived calvarial osteoblasts. Analysis of apoptosis in FOb and POb undergoing differentiation showed a significant difference, with the latter having more apoptotic activity. Apoptosis is an important determinant of the life-span of cells in regenerating tissue. During the process of bone regeneration, ∼50–70% of osteoblasts undergo apoptosis. Apoptosis is a naturally occurring cell death pathway induced in a variety of cell types and is associated with caspase activation or caspase mediation [18]. Caspase-3 is the most important protease in the caspase family, playing a pivotal role in apoptosis, cell survival, and osteogenic differentiation. Our results demonstrated significant differences in caspase-3 activity between FOb and POb undergoing to osteogenic differentiation, with elevated level in POb as compared to FOb cells. The higher activity of caspase-3 in POb was conversely paralleled by increased Bcl-2 expression in FOb, as revealed by fluorescent staining. It is known that Bcl-2 promotes cell survival [19] moreover, this antiapoptotic molecule plays important role in maintaining skeletal integrity [20].

Many factors orchestrate the pro- and anti-apoptotic activity in cells, including proteins in apoptotic pathway such as p53, Hsp70, granulocyte colony-stimulating factor, as well as chemicals or drugs, hypoxia, mechanical factors and activation of specific signaling pathways [21], [22], [23], [24], [25], [26]. Among the apoptosis regulatory signaling pathways, TGF-β, BMP and Wnt play important roles [11], [13], [14], [17], [27], [28], [29], [30], [31], [32], [33], [34]. Therefore, we investigated whether these three signaling pathways could play a role in determining differences in apoptosis between FOb and POb cells. Because our previous studies unveiled differential activation of endogenous FGF and Wnt signaling in FOb, POb and frontal and parietal bones as well [2], [3], [5], in the current study our experimental approach was first to determine whether difference in the activation of endogenous TGF-β signaling could be identified between FOb and POb cells. We then moved forward to investigate the potential role of this signaling pathway in apoptotic differences between FOb and POb. Activation of TGF-β signaling analyzed by endogenous phosphorylation of Smad-2 clearly indicated higher activation of TGF-β signaling in POb cells. This profile of activation was opposite from the endogenous profiles of FGF and Wnt signaling, previously observed in FOb and POb, with FOb displaying enhanced activation of both FGF and Wnt signaling compared to POb cells [2], [3], [5]. Thus, TGF-β signaling represents the first signaling pathway, among those we analyzed to be more activated in POb than FOb. TGF-β induced apoptosis has been widely reported in many tissues including epithelial, liver, endothelial, nervous, osteoblasts and mesenchymal cells [11], [12], [31], [32], [35], [36]. TGF-β signaling can play a dual role in apoptosis, either by upregulating or downregulate it, depending on the cell type and stimulation context [12], [37]. Our data showed that higher activation of TGF-β signaling in POb, at early stages of the osteogenic differentiation correlated with increased apoptosis in POb compared to FOb. Importantly, the same signaling pattern was also observed endogenously in FOb and POb, prior differentiation, indicating that the differential level of apoptotic activity and activation of TGF-β is part of their innate properties.

TGF-β induced apoptosis is mainly mediated through activation of Smads [13], [38], [39]. Our results indicate that indeed, TGF-β induced apoptosis in POb is mediated by activation of Smad-2. In fact, by using the TGF-β signaling inhibitor SB431542 we significantly decreased phosphoSmad-2 and apoptosis in POb. This result is in agreement with a previous study showing that inhibition of TGF-β pathway decreased apoptosis [11].

In turn, the inhibitory effect on apoptosis achieved by SB431542 treatment induced more robust osteogenesis on POb. Interestingly, TGF-β promoted apoptosis in FOb cells more prominently than in POb, while the inhibition by SB431542 was more prominent in POb. These observations suggest that blocking TGF-β signaling at certain stage of differentiation can be beneficial to improve the osteogenic capacity of mesodermal derived osteoblasts.

BMPs, other members of the TGF family, with strong pro-osteogenic activity [29], [40], [41], [42], [43], [44], [45], [46], also play an important role in apoptosis. Likely TGF-β, these molecules can either inhibit or induce apoptosis [27], [28], [47]. A study by Macias et al. suggested that BMP-2 and BMP-7 act as potent apoptotic signals for mesodermal cells, but not for ectodermal cells [29]. Interestingly, while TGF-β interfered with osteogenesis by activating kinase-1 (TAK1) and abrogating TAK1-mediated apoptosis [39], the activation of TAK1-p38 kinase pathway by BMP-2 induced apoptosis [14]. These observations implicate an important role for the interaction between TAK1 and Smad proteins on apoptosis and osteogenic development.

For the first time, we observed enhanced activation of BMP signaling in FOb compared to POb and its role in protecting FOb from apoptosis. Treatment with noggin, an inhibitor of BMP signaling increased apoptosis in FOb, while BMP-2 significantly decreased apoptosis in POb cells.

Because differential activation of canonical Wnt signaling in FOb and POb plays an important role in determining substantial differences in their osteogenic potential, as well as in repair of frontal and parietal bones [2], [48], we concluded our study by investigating whether canonical Wnt signaling would also contribute to the differential apoptotic activity observed between FOb and POb. Our findings indicate that indeed this signaling pathway is also a key player in determining differential apoptotic activity between FOb and POb. These evidences were provided by experiments performed either in presence of inhibitors or inducers of Wnt signaling. Treatment with Dkk1 and sFRP, inhibitors of Wnt signaling, dramatically increased apotosis in cells while decreasing osteogenic differentiation. Conversely, stimulation with Wnt3a decreased apoptosis. These findings are in agreement with previous reports showing that either inhibition or dysregulation of canonical Wnt signaling induced apoptosis [49], [50], [51].

**Figure 6:**
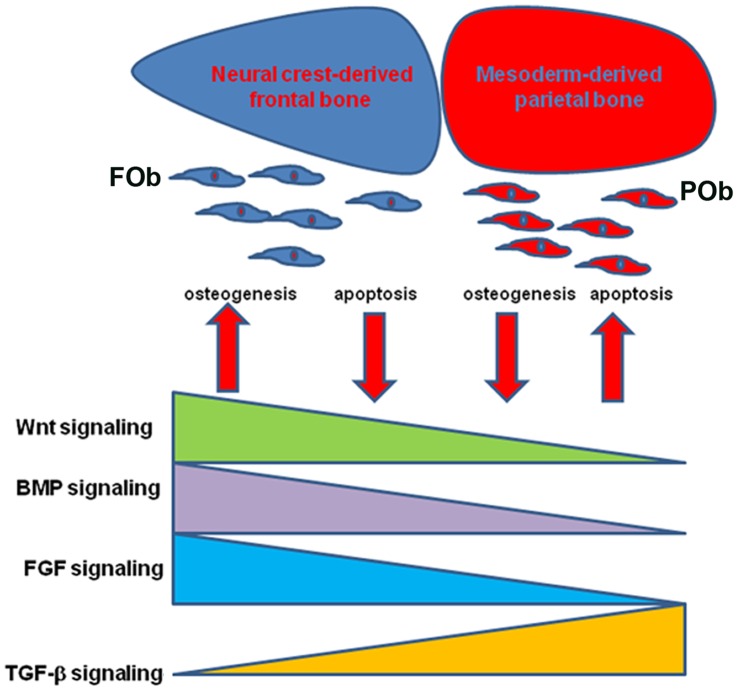
Proposed Model. Cartoon depicting the effect of integration of multiple signaling pathways, with differential activation, on neural crest-derived and mesoderm-derived bones and corresponding osteoblasts. The differential activation of these signaling(s) promotes greater osteogenesis and less apoptosis in FOb, and viceversa, less osteogenesis and higher apoptosis in POb.

Taken together, our results indicate that in FOb and POb integration of multiple signaling pathways having differential degree of activation orchestrate a signaling network which tightly controls apoptotic events and osteogenesis in FOb and POb (**Figure 6**).

Several molecular studies have demonstrated relationships between these signaling we have analyzed in FOb and POb. For instance, BMP and Wnt/β-catenin signaling pathways cooperatively regulate osteoblast differentiation and bone formation. Zhang et al. demonstrated that Wnt/β-catenin and BMP signaling pathways tightly cooperate and regulate each other [52]. Furthermore, Wnt/β-catenin signaling is an upstream activator of BMP-2 expression, and blocking this pathway decreased BMP-2 expression in osteoblasts [52]. Moreover, activation of β-catenin induces osteoblasts differentiation and participates in BMP mediated signal transduction [53]. Bain and collegouses showed that BMP induced differentiation of osteoblasts leading to endogenous activation of β-catenin canonical Wnt signaling [53]. Interplays between FGF-2, BMP and Wnt signaling have also been reported [2], [54], [55]. Indeed, we have previously demonstrated that enhanced activation of Wnt signaling in FOb is mediated by FGF signaling through GSK-3β inhibition [2]. Moreover, a cross-talk between TGF-β and BMP signaling pathways, in the context of bone differentiation has been shown [56], [57], [58]. Therefore, it will be of interest to investigate whether there is a cross-talk between these signaling pathways in FOb and POb cells.

The rate of bone formation is largely determined by the number of osteoblasts, which in turn is determined by the rate of replication of progenitors and the life-span of mature cells, reflecting the timing of death by apoptosis. Because of evidence that apoptosis is the fate of the majority of osteoblasts, changes in the prevalence of osteoblast apoptosis should alter the rate of bone formation. This is in line with our previous and current observation, indicating that FOb proliferate more than POb cells [3], [4], [5] and have less apoptotic activity than POb. Taken together, our findings provide a compelling explanation for the greater osteogenic capacity of neural crest-derived compared to mesoderm-derived osteoblasts.

## Materials and Methods

### Animals

All experiments using animals were performed in accordance with Stanford University Animal Care and Use Committee guidelines (protocol ID #8397). The Institutional Animal Care and Use Committee (IACUC) specifically approved this study. CD-1 wild-type mice were purchased from Charles River Laboratories Inc., Wilmington, MA. Animals were housed in light- and temperature-controlled rooms and were given food and water *ad libitum*.

### Tissue Harvesting and Primary Cell Culture

Neural crest-derived frontal osteoblasts (FOb) and mesoderm-derived parietal osteoblasts (POb) were harvested from skulls at embryonic day 17.5 (E17.5), 18.5 (E18.5) and postnatal day 1 (P1), day 7 (P7), day 30 (P30) and day (P60), respectively as previously described [59]. The periosteum and dura mater were carefully stripped off from the skull. The peripheral suture complexes of each frontal or parietal bone were also carefully removed. Frontal and parietal bones were minced separately into small chips less than 1 mm before digestion. Embryonic skulls were digested with 0.1% Collagenase A (Roche Diagnostics, Indianapolis, IN, USA) in serum free alpha-minimum essential medium (α-MEM), (Gibco Life Technologies and Invitrogen Corporation, Carlsbad, CA) for 30 minutes at 37°C in a shaking water bath.

Postnatal skulls were digested with 0.2% Dispase II and 0.1% Collagenase A (Roche Diagnostics, Indianapolis, IN, USA) in serum-free medium. The digestion was carried out 6 times, each 10 minutes. The first two digestions were discarded. The later four digestions were pooled together. All digestions were neutralized with an equal volume of α-MEM supplemented with 10% fetal calf serum (FCS), (Gemini Bioproducts, Woodland, CA), 100 IU/ml penicillin and streptomycin (Gibco Life Technologies and Invitrogen Corporation, Carlsbad, CA), pelleted and resuspended in the growth medium. Both FOb and POb cells were plated in 100 mm tissue culture dishes (Corning Incorporated, New York, NY) and incubated at 37°C with continuous supplement of 5% CO_2._ The medium was changed every other day. Only passage 0 and 1 cells were used for all experiments.

### Osteogenic Differentiation Assay

FOb or POb were plated in 6-well-plate (1×10^5^/well). Upon sub-confluence, cells were incubated with the osteogenic differentiation medium (ODM), composite of α-MEM supplemented with 10 µM glycerol β-phosphate, 0.25 µM ascorbic acid, (Sigma Aldrich, St. Louis, MO), 10 or 1% FCS, and 1% penicillin/streptomycin. The medium was changed every other day. Where requested differentiation assays were also performed in presence of Ac-DEVD-CHO (0.05 µM), TGF-β1 (10 ng/ml), BMP-2 (200 ng/ml), Wnt (50 ng/ml) TGF-β signaling inhibitor SB431542 (10 µM), BMP signaling inhibitor noggin (200 ng/ml), and Wnt signaling inhibitors Dkk-1 and sFRP-1 (150 ng/ml for each). All reagents were purchased from R&D Systems, (Minneapolis, MN). Mineralization of the extracellular matrix was assessed by Alizarin Red staining at day 21 of the differentiation followed by its quantification as previously described [2], [48]. All morphological observations and analysis were conducted by using Leica DMIL microscope and Leica Microsystems digital imaging software (Leica Microsystems Wetzlar, Germany).

### Measurement of Caspase-3 Activity

The caspase-3 fluorometric protease assay was performed using a Caspase-3 Apoptosis Detection Kit (sc-4263 AK, Santa Cruz Biotechnology) according to the manufacturer’s instructions and previously described protocols [60], [61], [62]. FOb and POb were cultured either in growth medium or ODM supplemented with or without either the inhibitor of apoptosis, Ac-DEVD-CHO, or each of specific stimulators and/or inhibitors of the different signaling pathways as described above. Cell lysates were collected at different time points, 40 µl of cell lysate (in triplicate) were incubated with 200 µl of reaction buffer, 5 µl of EDVD-AFC substrate and DTT (final concentration of 10 mM). The reaction mixtures were incubated at room temperature for 1 hour. The analysis was conducted using a fluorescent microplate reader (SpectraMAX Gemini XS, Molecular Devices Corporation, CA, USA) at excitation/emission wavelength of 400/505 nm. Levels of emission of FOb and POb were compared.

### Indirect Immunofluorescent Staining

FOb and POb were seeded on circle glass coverslips (12 mm) in triplicate and placed in 6 well plates with growth medium (α-MEM, 10% FBS, 1% penicillin and streptomycin). After 48 hours cells were washed twice with Phosphate buffered saline (PBS) and fixed with 50% acetone-50% methanol for 20 minutes at 4**°**C, followed by washing with PBS-0.1%Triton-100 twice. Then, cells were incubated in a blocking solution with 1% horse serum in PBS-0.05% Tween-20 for 1 hour at RT followed by incubation with primary antibody overnight at 4**°**C. Primary antibodies used were as follows: rabbit anti-phospho-Smad1/5 (Ser463/465) antibody (1∶50 dilution); rabbit anti-phospho-Smad2 (Ser465/467) antibody (1∶50 dilution) and rabbit anti-phospho-Bcl-2 antibody (1∶100 dilution), all purchased from Cell Signaling (Danvers, MA). After, cells were washed three times with PBS/0.1% Tween-20 and incubated in the blocking solution for 1 hour at room temperature followed by incubation with fluorescein-conjugated donkey anti-rabbit IgG secondary antibody, Alexa-fluor 488, and Alexa-fluor 555 (dilution 1∶800), (Molecular Probes, Invitrogen, Carlsbad, CA) for 1 hr at room temperature. Nuclear counterstaining was performed using Vectashield H-1200 mounting medium with DAPI (Vector Laboratories, Burlingame, CA). A Zeiss Axioplan microscope equipped with an Axiocam HRc digital camera was used for imaging.

### Immunoblotting Analysis

FOb and POb were collected at different time points of osteogenic differentiation assay performed with or without stimulators and/or inhibitors of TGF-β, BMP and Wnt signaling. Cells were lysated with cold RIPA buffer (50 mmol/L of HEPES, pH 7.5, 150 mmol/L of NaCl, 1 mmol/of EDTA, 10% glycerol, 1% Triton-X-100, 25 mM sodium fluoride) containing 1 mM sodium orthovanadate and Proteases Inhibitor Cocktail (Sigma-Aldrich, St. Louis, MO). Cell lysates (40 µg) were electrophoresed on 12% Tris-HCl sodium dodecyl sulfate (SDS)-PAGE gels (Precast Nupage gels, Gibco Life Technologies and Invitrogen Corporation, Carlsbad, CA) and transferred onto Immobilon-P membrane (Millipore Corporation, Bedford, MA). Immunoblotting analysis was performed using the following primary rabbit antibodies: anti-phosphorylated Smad-2 (Ser465/467), anti-Smad-2, anti-phosphorylated Smad-1/5 (Ser465/467), anti-Smad-5 (1∶1000; Cell Signaling Danvers, MA), anti-active β-catenin (anti-ABC), clone 8E7 (1∶4000; Millipore, Tamecula, CA), anti-β-catenin (1∶500; Millipore, Tamecula, CA) and anti-α-tubulin (1∶6000; ab8227, Abcam, Cambridge, MA). A horseradish peroxidase-conjugated secondary anti-rabbit was used (1∶2000; Cell Signaling Danvers, MA). Immunoblotted proteins were visualized by enhanced chemiluminescence (Amersham Biosciences, Buckinghamshire, UK). To assess for the total amount of endogenous Smad-2 and Smad-1/5, and to control for equal loading and transfer of the samples the membranes were reprobed with anti-Smad-2, anti-Smad-1/5 antibodies and anti-α-tubulin antibody. Densitometry analysis of electrophoretic bands was performed using the ImageJ software program, (NIH, Bethesda, MA). The density of pSmad-2 and pSmad1/5 bands were normalized to the loading controls (α-tubulin) and presented as percentage increase. The results are the mean ±SD of three independent experiments.

### Histology and TUNEL Assay

Following sacrifice of mice by CO_2_ asphyxiation, the calvariae were harvested, dissected and immediately fixed in fresh 10% neutral buffered formalin overnight. Subsequently the calvarial were decalcified in 19% EDTA at 4°C for the appropriate time. Following decalcification, specimens were embedded in paraffin and cut into 8 µm sections. The apoptosis TUNEL assay was performed by labeling the fragmented DNA with an apoptosis detection kit according to the manufacturer’s instructions (ApoTag Plus Peroxidase *In Situ* Apoptosis Detection Kit, Millipore, Temecula, CA 92590, USA). Sections were examined with Carl Zeiss Axioplan 2 (Zeiss, Thornwood, NY) microscope from at 20× magnification. Images were captured by AxioVision (Zeiss, Thornwood, NY) and combined by Adobe Photoshop (Adobe Systems, San Jose, CA).

### Statistical Analysis

Data are expressed as mean ±SD of three independent experiments. The error bars in the graphs represent one standard deviation. Statistical differences between the means are examined by Student’s test. A *P* value <0.05 was considered statistically significant.

## Supporting Information

Figure S1 **Direct inhibition of apoptosis increases the osteogenic potential of POb.** (A)Treatment with the specific inhibitor of apoptosis AC-DEVD-CHO dramatically decreases the apoptotic activity in POb cells. (B) Alizarin red staining of FOb and POb cells shows a significant increase of osteogenic differentiation at day 21 in treated POb cells, to a level similar to that of untreated FOb. (Magnification 10X). (C) Quantification of alizarin red staining.
(TIF)

Figure S2 ***In vivo*** **apoptosis profile of Frontal and Parietal bones. (A)**TUNEL analysis performed on coronal sections obtained from frontal and parietal bones at postnatal day 7. Numerous apoptotic TUNEL-positive osteoblasts (arrowheads) are observed in the parietal bone plate as compared to frontal bone plate. (B) A similar apoptotic pattern is revealed by TUNEL assay in postnatal day 30 frontal and parietal bones. (Magnification at 20X). Abbreviations: PO, periostium; DM, dura-mater; BP, bone plate; P, postnatal. (C) Quantification of TUNEL-positive osteoblasts. Asterisks indicates significant differences with value *P<0.05.* Scale bar: 100 µm.
(TIF)
