# Supplementary figures and images for: Integration of Multiple Signaling Regulates through Apoptosis the Differential Osteogenic Potential of Neural Crest-Derived and Mesoderm-Derived Osteoblasts
Source: PLoS One. 2013 Mar 25;8(3):e58610. doi: 10.1371/journal.pone.0058610 (PMC3607600; doi:10.1371/journal.pone.0058610)

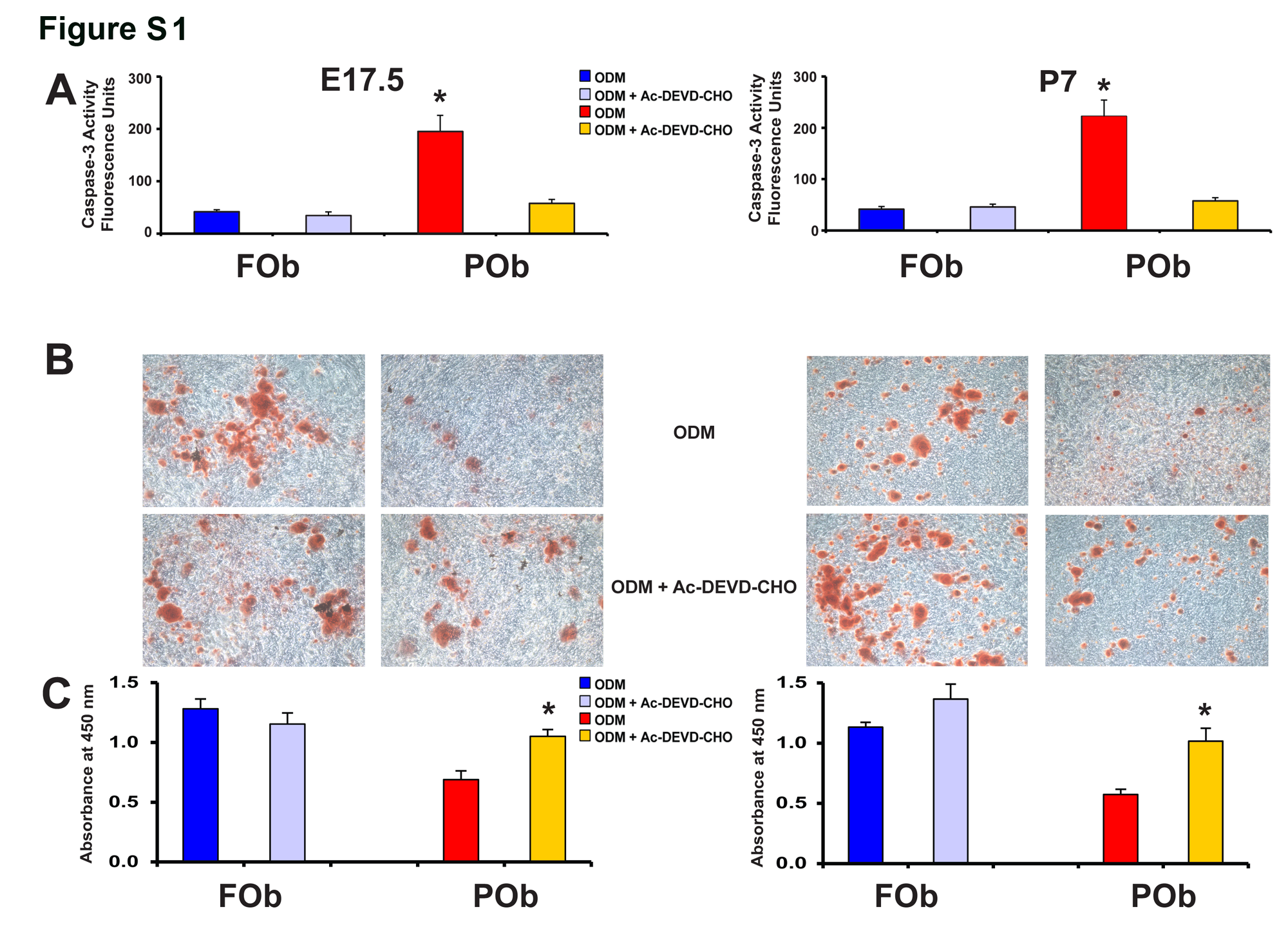

Supplement: Figure S1 — Direct inhibition of apoptosis increases the osteogenic potential of POb. (A)Treatment with the specific inhibitor of apoptosis AC-DEVD-CHO dramatically decreases the apoptotic activity in POb cells. (B) Alizarin red staining of FOb and POb cells shows a significant increase of osteogenic differentiation at day 21 in treated POb cells, to a level similar to that of untreated FOb. (Magnification 10X). (C) Quantification of alizarin red staining. (TIF) [file pone.0058610.s001.tif]

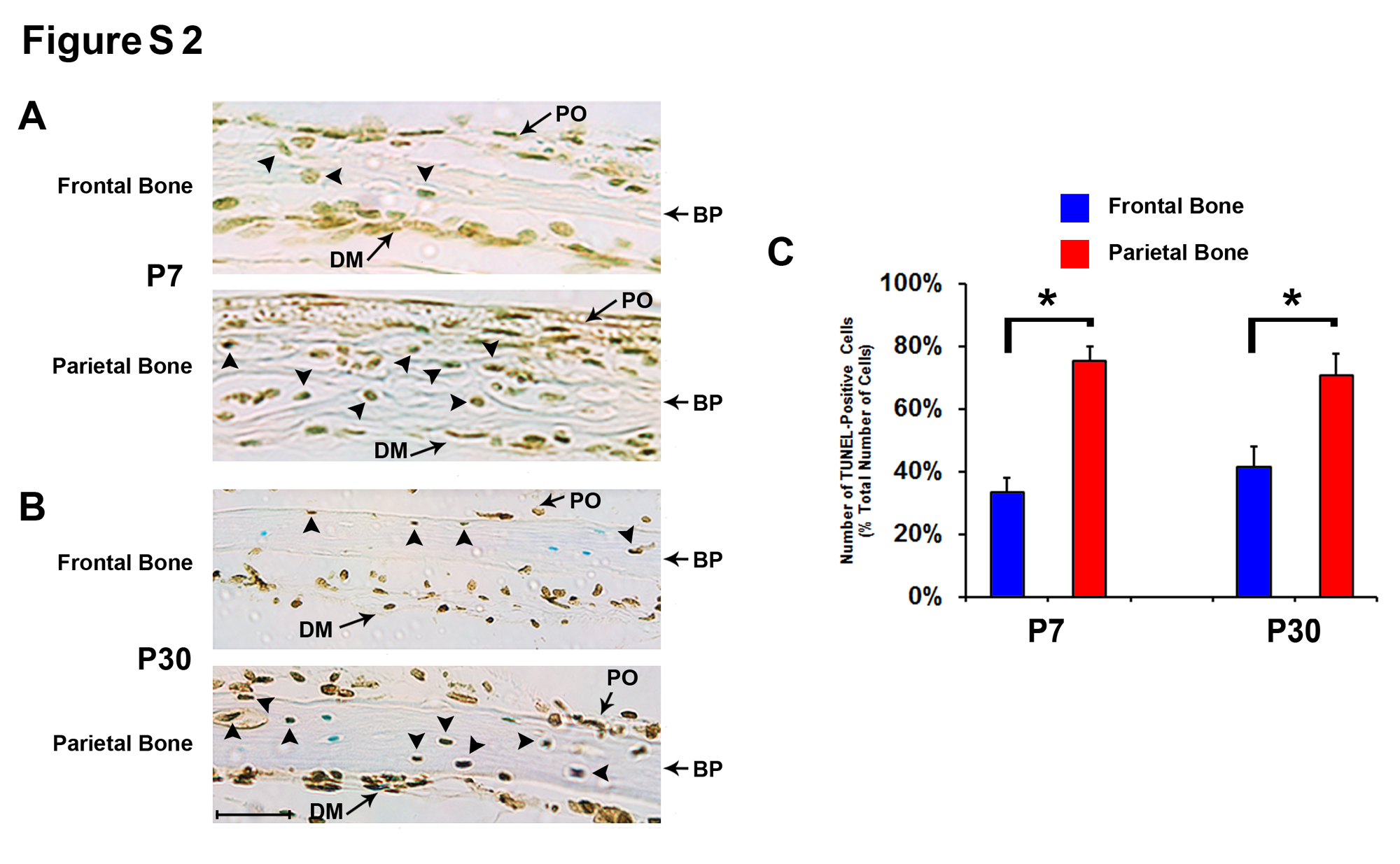

Supplement: Figure S2 — In vivo apoptosis profile of Frontal and Parietal bones. (A)TUNEL analysis performed on coronal sections obtained from frontal and parietal bones at postnatal day 7. Numerous apoptotic TUNEL-positive osteoblasts (arrowheads) are observed in the parietal bone plate as compared to frontal bone plate. (B) A similar apoptotic pattern is revealed by TUNEL assay in postnatal day 30 frontal and parietal bones. (Magnification at 20X). Abbreviations: PO, periostium; DM, dura-mater; BP, bone plate; P, postnatal. (C) Quantification of TUNEL-positive osteoblasts. Asterisks indicates significant differences with value P<0.05. Scale bar: 100 µm. (TIF) [file pone.0058610.s002.tif]
